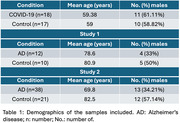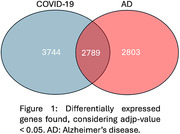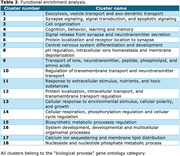# Transcriptomic similarities in the brain of Alzheimer’s disease and COVID‐19 patients

**DOI:** 10.1002/alz.095193

**Published:** 2025-01-09

**Authors:** Guilherme Bastos de Mello, Giovanna Carello‐Collar, Débora Guerini de Souza, Marco Antônio de Bastiani, Eduardo R. Zimmer

**Affiliations:** ^1^ Pontifícia Universidade Católica do Rio Grande do Sul, Porto Alegre, Rio Grande do Sul Brazil; ^2^ Universidade Federal do Rio Grande do Sul, Porto Alegre, RS Brazil; ^3^ Federal University of Rio Grande do Sul (UFRGS), Porto Alegre, RS Brazil; ^4^ Federal University of Rio Grande do Sul, Porto Alegre, Rio Grande do Sul Brazil; ^5^ Universidade Federal do Rio Grande do Sul, Porto Alegre Brazil

## Abstract

**Background:**

Neurological symptoms, such as the lack of concentration, memory impairment and cognitive dysfunction are among the main complaints of COVID‐19 patients and seem to overlap with some of the Alzheimer’s disease (AD) symptoms. However, whether COVID‐19 and AD share a molecular signature is yet unclear. Thus, we aimed to explore transcriptomic similarities in the brains of COVID‐19 and AD individuals.

**Method:**

We obtained frontal cortex transcriptomics datasets of COVID‐19 and AD from the Gene Expression Omnibus. Demographic characteristics of included individuals are reported in **Table 1**. Differentially expressed genes (DEGs) of COVID‐19 or AD versus controls were identified using the R statistical environment (FDR‐adjusted p‐value <0.05). Functional enrichment analyses (FEA) were accomplished to identify similar signatures considering the overlapped DEGs found between these two conditions.

**Result:**

We identified 6,533 DEGs in COVID‐19 brains and 5,592 in AD brains. We found a striking overlap of 2,789 DEGs between these pathologies (**Figure 1**). Considering the DEGs in common, cluster observation suggests that COVID‐19 and AD share similar enriched processes, such as “central nervous system differentiation and development”, “signal release from synapse and neurotransmitter secretion” and “cognition, behavior, learning and memory” (**Table 2**).

**Conclusion:**

Our results highlight core molecular programs shared by COVID‐19 and AD in the frontal cortex. These similarities may contribute to understanding the pathogenesis and the clinical course of COVID‐19 neurologic manifestations. These findings highlight the need for a careful evaluation of COVID‐19 survivors still presenting with neurological symptoms, which could be at high risk of developing AD.